# IIb‐RAD‐sequencing coupled with random forest classification indicates regional population structuring and sex‐specific differentiation in salmon lice (*Lepeophtheirus salmonis*)

**DOI:** 10.1002/ece3.8809

**Published:** 2022-04-06

**Authors:** Prashanna Guragain, Anna Solvang Båtnes, John Zobolas, Yngvar Olsen, Atle M. Bones, Per Winge

**Affiliations:** ^1^ 8018 Cell, Molecular Biology and Genomics Group Department of Biology Norwegian University of Science and Technology Trondheim Norway; ^2^ 8018 Taskforce Salmon Lice Department of Biology Norwegian University of Science and Technology Trondheim Norway

**Keywords:** geographical distribution, Norway, population genetics, salmon lice, sex

## Abstract

The aquaculture industry has been dealing with salmon lice problems forming serious threats to salmonid farming. Several treatment approaches have been used to control the parasite. Treatment effectiveness must be optimized, and the systematic genetic differences between subpopulations must be studied to monitor louse species and enhance targeted control measures. We have used IIb‐RAD sequencing in tandem with a random forest classification algorithm to detect the regional genetic structure of the Norwegian salmon lice and identify important markers for sex differentiation of this species. We identified 19,428 single nucleotide polymorphisms (SNPs) from 95 individuals of salmon lice. These SNPs, however, were not able to distinguish the differential structure of lice populations. Using the random forest algorithm, we selected 91 SNPs important for geographical classification and 14 SNPs important for sex classification. The geographically important SNP data substantially improved the genetic understanding of the population structure and classified regional demographic clusters along the Norwegian coast. We also uncovered SNP markers that could help determine the sex of the salmon louse. A large portion of the SNPs identified to be under directional selection was also ranked highly important by random forest. According to our findings, there is a regional population structure of salmon lice associated with the geographical location along the Norwegian coastline.

## Graphical abstract

The combination of IIb‐RAD sequencing and random forest enables the discovery of SNP markers for the population structure and sex determination. Our data suggest that salmon lice have a regional population structure associated with their geographic position along the Norwegian coast. Additionally, the sex of lice can be deduced by using marker SNPs.

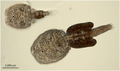

## 1 INTRODUCTION

The genetic assignment of individuals to their reference population is valuable to recognize the spatial distribution of populations and their migration patterns (André et al., 2016). Assignment tests using individual genotypes to classify individuals or populations into clusters have been evaluated and applied in various marine species such as Atlantic salmon, *Salmo salar* (Gilbey et al., 2018; Glover et al., 2008; Jeffery et al., 2018), Chinook salmon, *Oncorhynchus tshawytscha* (Dehaan et al., 2018; Meek et al., 2016; Templin et al., 2011), Atlantic cod, *Gadus morhua* L. (André et al., 2016; Berg et al., 2016), and herring, *Clupea harengus* (Bekkevold et al., 2015). Using these genetic assignment tests in other marine organisms may help to detect divergence in their populations.

**FIGURE 1:**
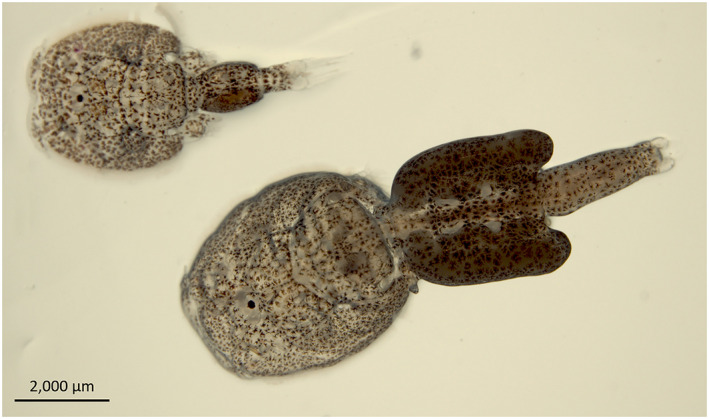
Salmon lice adult life stage. Salmon louse adult male (above) and adult female (below). Adult female produces egg strings (not shown in the figure)

There is great interest in the population genetic assignment of salmon louse (*Lepeophtheirus salmonis*) along the Norwegian coastline. Figure 1 depicts an adult male and adult female salmon louse. Salmon lice feed on mucus, blood, and epidermal tissues, and they have a significant effect on salmonids during the marine part of their lifecycle (Costello, 2006; Johnson et al., 2004). Higher infestation rates result in skin lesions and secondary microbial and viral infections, and in elevated mortality in the absence of delousing (Grimnes & Jakobsen, 1996; Guragain, Tkachov, et al., 2021; Pike & Wadsworth, 1999). Salmon lice infestations have a substantial economic burden on the Norwegian aquaculture industry and are anticipated to increase in the coming years (Liu & Bjelland, 2014). Increased costs of production related to salmon lice control include non‐medical treatments, chemotherapeutic treatments, the buying and maintenance of cleaner fish, net cleaning, loss of salmon due to mortality, and handling costs and higher feed consumption ratios (Guragain, Tkachov, et al., 2021; Iversen et al., 2018). The need for a comprehensive study of these ectoparasites was triggered by the huge economic loss and fish welfare issues, which are still ongoing to the present day (Costello, 2009; Heuch & Mo, 2001).

A key aspect of assessing infestation, connectivity, and the spread of salmon lice and associated drug resistance alleles is recognizing and predicting salmon louse dispersal. Several attempts have been made to classify the genetic structure in salmon louse in the North Atlantic. Population structure or population stratification is caused by nonrandom mating between individuals, which results from the isolation of subpopulations with low rates of migration and gene flow across multiple generations (Hellwege et al., 2017). High gene flow between various locations has frequently been detected using standard methods such as microsatellite and sequencing markers with no indication of population structure (Glover et al., 2011; Nolan & Powell, 2009; Todd et al., 2004). A similar outcome was published with a genome‐wide SNP array of 5,091 variable markers suggesting a single panmictic lice population in the Atlantic Ocean (Besnier et al., 2014). These reports show that the detection of population structure in salmon louse has made significant progress, but these methods remain costly and difficult. The emergence of high throughput next‐generation sequencing (NGS) approaches, the use of restriction enzyme on site‐associated DNA sequencing (RAD‐seq) enables a small fraction of the genome to be sequenced across multiple samples (Davey & Blaxter, 2010). The benefits of this method include simplicity and cost‐efficiency. Recently, IIb‐RAD sequencing coupled with a random forest classification approach was used to identify the essential SNPs for the fine‐scale differentiation of salmon louse populations in the North Atlantic. The method identified 98 discriminatory SNPs that improved the population assignment, indicating that it can differentiate among nearby salmon louse populations using highly specific SNPs (Jacobs et al., 2018). Understanding the gene flow and connectivity of salmon lice in Norway is critical for selective treatment interventions, and observations of North Atlantic salmon lice populations possessing common gene mutations for insecticides have been reported (Aaen et al., 2015; Besnier et al., 2014; Messmer et al., 2018; Todd et al., 2004).

Insect sex determination mechanisms have provided us with valuable insights into genetics and how these molecular mechanisms have evolved over time (Sánchez, 2004). The genotype of the zygote determines sex in the majority of insects. Male heterogamety, female heterogamety, haplodiploidy, and paternal genome elimination are examples of common sex determination systems (Blackmon et al., 2017). According to reports, the salmon lice genome is consistent with a female heterogamety containing both ZZ‐ZW and ZZ‐Z0 sex chromosomes (Danzmann et al., 2019; Skern‐Mauritzen et al., 2021). At the early stages of development, salmon lice lack distinguishable secondary sex features, therefore a sex‐specific marker is valuable for early sex identification. RAD‐seq has been used to classify a sex‐specific marker and characterize sex determination in salmon louse (Carmichael et al., 2013). Here, we use IIb‐RAD‐seq coupled with random forest classification to identify additional new markers associated with sexual differentiation.

We set out to investigate the salmon lice genetic variations along the Norwegian coast and to identify markers for population structure and sexual differentiation. Samples were collected from 12 different farming locations in various aquaculture production areas along the Norwegian coast (Ministry of Trade, Industry, & Fisheries, 2015). The IIb‐RAD‐based method was employed to produce SNP data and machine learning algorithms were used to derive the salmon louse population structure.

## 2 MATERIALS AND METHODS

### 2.1 Sample collection and gDNA extraction

**FIGURE 2:**
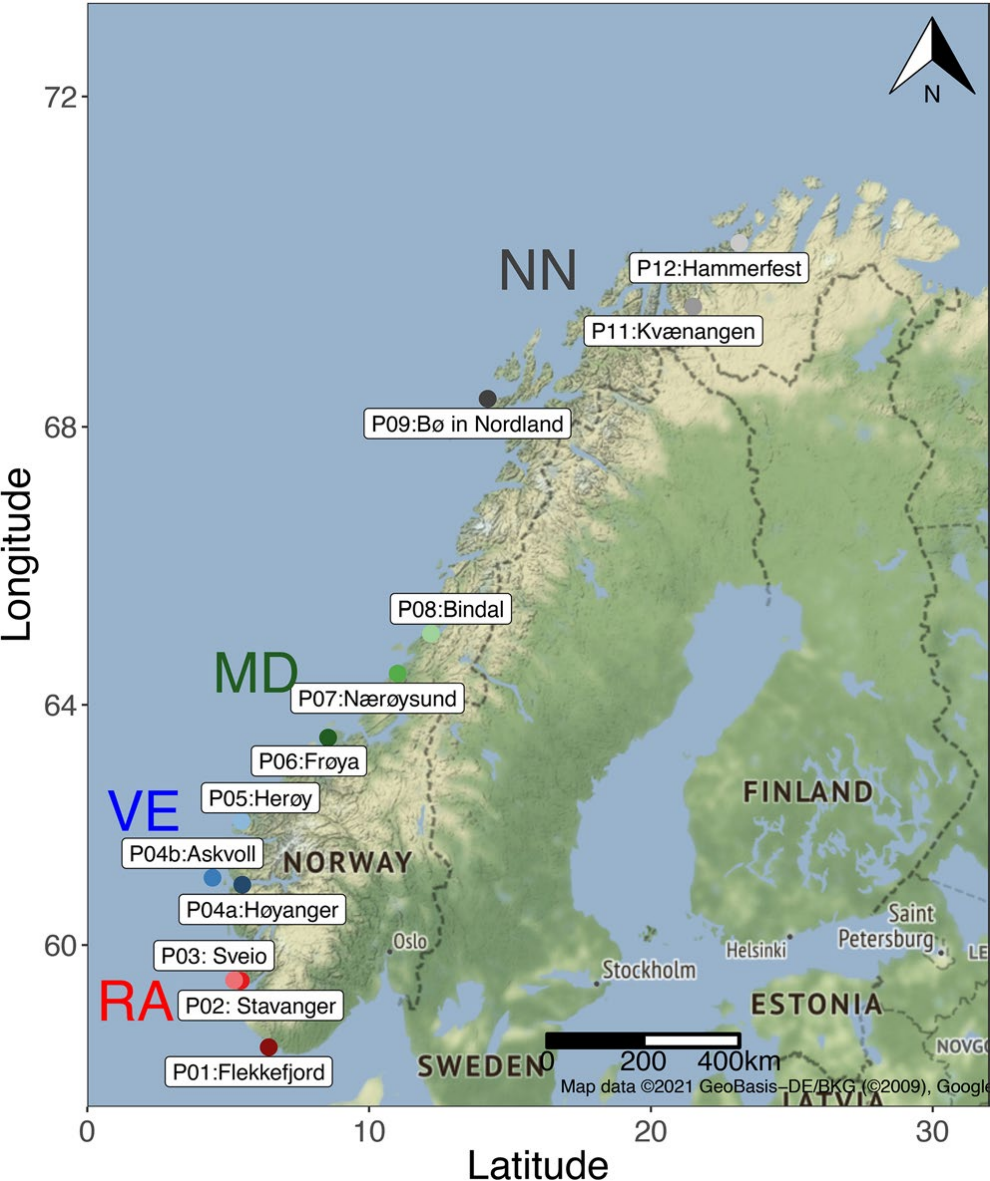
Geographical locations of lice sampling. The map shows all 12 sites of salmon lice collection. Each 3 sampling sites are grouped regionally RA: P01‐P03, VE: P04a‐P05, MD: P06‐P08, and NN: P09‐P12. The coordinates and full details of locations are included in Table S1. Map was plotted using R package *ggmap*

Lice (*L*. *salmonis*) samples were collected from 12 different geographical locations along the Norwegian coast from commercial Atlantic salmon pens in 2019 (Table S1). A total of 95 salmon lice individuals were collected (Table 1) and sites include locations from Finnmark in the North to Agder in the South (Figure 2). Pre‐adult females and males were selected for IIb‐RAD sequencing to prevent contamination of the gametes. DNA was isolated using Qiagen DNeasy Blood & Tissue Kits (Qiagen, Halden, Germany) and quantified using NanoDrop™ One spectrophotometer. Quantified DNA was visualized on a 1% (w/v) quality agarose gel and DNA extracts from each site were selected for further analysis.

**TABLE 1:** Summary statistics, namely observed heterozygosity (Ho), expected heterozygosity (Hs), and coefficient of inbreeding (Gis)

			Full SNPs			Rf‐reduced SNPs (RfGeo)		
Poparea	Regional geography	*N*	Ho	Hs	Gis	Ho	Hs	Gis
P01	RA	8	0.181	0.185	0.022	0.242	0.262	0.075
P02		7						
P03		8						
P04a	VE	8	0.188	0.187	−0.003	0.253	0.267	0.053
P04b		8						
P05		8						
P06	MD	8	0.195	0.19	−0.029	0.251	0.261	0.038
P07		8						
P08		8						
P09	NN	8	0.179	0.183	0.024	0.246	0.267	0.078
P11		8						
P12		8						

Note

Genetic diversity was calculated using full‐SNP dataset and RfGeo dataset.

### 2.2 Library preparation and sequencing

Library preparation was performed as described in (Wang et al., 2016). For each sample, 100–200 ng genomic DNA was digested by 1 U BsaXI (NEB, #cat R0609) to produce fragments of uniform length. The effectiveness of digestion was verified on 1% (w/v) agarose gel. Digested DNA was ligated with five labels of standard 5′‐ NNN‐3′ adaptors to ligate with restriction tags. The amplified products were connected in series according to the five sets of adaptors and gel purified. Barcodes were introduced by PCR with barcode‐bearing primers. PCR products were purified using a MinElute PCR Purification Kit and pooled for sequencing. The libraries were sequenced in Illumina NovaSeq platform by using paired‐end sequencing configuration (CD Genomics, USA).

### 2.3 Data processing

Using digital digestion, the tags containing the restriction enzyme recognition sites were extracted from the reference genome as the reference sequence. Short paired‐ends were aligned to a reference sequence using SOAP software (version 2.21) (Li et al., 2009), and SNP calling was performed using the maximum likelihood estimate of the allele frequency (Hohenlohe et al., 2010). The RADtyping software package (Fu et al., 2013) was used throughout the entire process, from data preprocessing to the final typed output. For the accuracy of the subsequent analysis, the data were filtered by removing sites containing only one genotype, sites where the genomic base is N, sites with more than 2 SNPs within the tag, sites where two different tags call different types within the same site, all loci where less than 80% of the sample can be typed, sites with minor allele frequency (MAF) of less than 0.01 and sites with alleles greater than 2. Software SnpEff (version:4.1g) (Cingolani et al., 2012) was applied to annotate the resulting SNP to determine the position of the SNP in the genetic element and the effect on the amino acid changes.

### 2.4 Population genomic analyses

Principal component analysis (PCA), discriminant analysis of principal components (DAPC), and population assignment probabilities were calculated using *adegenet* (Jombart & Ahmed, 2011). Genetic diversity, namely observed heterozygosity (Ho), expected heterozygosity (Hs), and coefficient of inbreeding (Gis), analyses of molecular variance (AMOVA) (Excoffier et al., 1992), population assignment, and pairwise difference were calculated using *genodive* (Meirmans, 2020). *p*‐values for fixation index (Fst) were false discovery rate (FDR) adjusted for multiple comparisons using the R package *p*‐*adjust*, which implements the Benjamini‐Hochberg step‐up procedure (Benjamini & Hochberg, 1995). Heatmap was created for the pairwise difference using R package *pheatmap v3*.*2*. Loci putatively under positive selection were identified in *Arlequin* (Excoffier & Lischer, 2010), *p*‐value adjusted using R package *p*.*adjust* and plotted using R package *ggplot2*. Neutral mutation hypothesis with Tajima's D was tested using R package *pegas* (Paradis, 2010). Isolation by distance was tested using the Mantels test (Diniz‐Filho et al., 2013) using R package *vegan*. Furthermore, we performed a second outlier analysis using *BayeScan* (Foll & Gaggiotti, 2008) with a prior odd of 10, as it has a lower type I error compared with *Arlequin*. R v4.4 was used for analyses done in R (R Core Team, 2020).

### 2.5 Use of Random Forest and UMAP to detect important SNPs and visualization

The SNP characteristics of each population were detected using a tree‐based ensemble machine‐learning method, the *randomForest* R package (Liaw & Wiener, 2002). Random forest is a supervised learning method based on the aggregation of a number of classification trees to build classification rules (Breiman, 2001). The populations were numerically encoded, and the missing data were imputed using the *rf*.*impute* command. The data were divided into training and test dataset. Three independent random forest runs were checked for convergence by performing a Pearson correlation between SNP importance values (Brieuc et al., 2018). 400,000 trees were used to tune the geographical importance of SNPs, whereas 25,000 trees were used for sex importance of SNPs. The SNP were ranked using the mean decrease in accuracy (MDA) parameter in the functional importance. The MDA is a decrease in the accuracy of the prediction rule due to the random permutation of the values in each feature (Hastie et al., 2009). For the backward purging approach, SNPs with negative MDA values were removed as nondiscriminatory and backward purging was performed on top‐ranked 2% of SNPs (Brieuc et al., 2018; Laporte et al., 2016). We determined the SNPs with the highest discriminatory power based on the lowest out of bag (OOB) error rate and used it in downstream analyses. Two datasets were created using the backward purging of SNPs based on regional geography (RfGeo), and sex (RfSex).

Uniform Manifold Approximation and Projection (UMAP) is a novel nonlinear dimension reduction method built on the theoretical foundations of Riemannian geometry and algebraic topology (McInnes et al., 2020). This method was used to reduce the full‐SNP dataset and Random Forest‐reduced (Rf‐reduced) dataset to 2 dimensions, suitable for visualizing and distinguishing clusters of various lice based on population area, regional geography and sex.

## 3 RESULTS

### 3.1 Processing of IIb‐RAD data and summary

An average of 10.7 ± 1.85 million reads was generated for 95 individuals from 12 sampling locations along the Norwegian coastline using IIb‐RAD sequencing. The final catalog contained 157,815 RAD tags with an average depth of 40.9 ± 9.8 per individual covering 0.61% of the genome (Figure S1). After stringent filtering, a total of 19,428 SNPs were retained.

### 3.2 Effect of SNP on the amino acid changes

The influence of the SNP on the proteins was evaluated, and nine highly affecting mutations that might cause destructive effects like protein truncation and loss of function were observed. Similarly, 340 missense and nonsynonymous mutations were also observed that could lead to nondestructive variation that may affect the efficacy of protein function. Six nonsense mutations that could lead to a stop codon resulting in premature termination of peptide chain synthesis have been observed. These effects are summarized in Table S2.

### 3.3 Identification of SNPs putatively under selection

**FIGURE 3:**
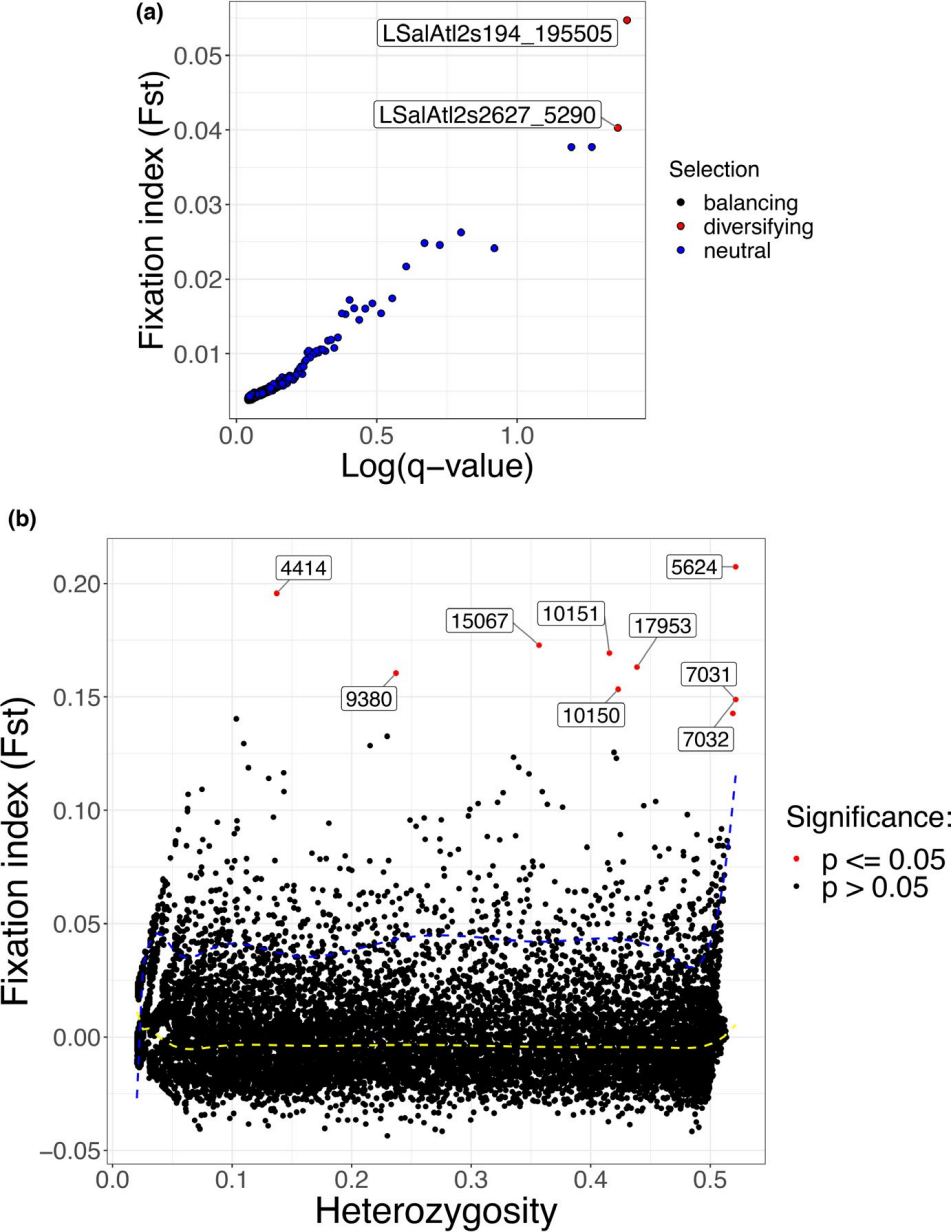
SNPs putatively under selection. (a) Output plot of BayeScan software where each dot represents one SNP out of 19428 SNPs. Black, red, and blue color represent balancing, diversifying, and neutral SNPs, respectively. The SNPs putatively under diversifying selection are annotated by contig number and position. (b) Detection of loci under selection from genome scans based on Fst simulations implemented in Arlequin. Red annotated dots represent loci under diversifying selection, and details of each locus under selection are listed in Table 3. Dotted yellow and blue lines indicate 50% and 95% quantiles, respectively

*BayeScan* identified 2 SNPs putatively under divergent selection, whereas 11,705 SNPs as potentially subject to balancing selection, and the remaining 7,721 SNPs were retained as putatively neutral (Figure 3a). Unlike *BayeScan*, *Arlequin* listed 9 SNPs under selection, while the remainder were putatively neutral. However, two SNPs LSalAtl2s194_195505 and LSalAtl2s2627_5290 were listed as “putatively under selection” by both software (Figure 3b).

### 3.4 Population structure using the full‐SNP dataset

The full dataset of 19,428 SNPs was used to test the genetic structure of the populations using different methods. Observed heterozygosity (H_o_) ranged from 0.152 to 0.209, while expected heterozygosity within population (H_s_) ranged from 0.176 to 0.196 (Table 1). For the majority of the sites, observed heterozygosity was found to be smaller than expected heterozygosity. G_IS_ values varied along the sample areas and were positive or near zero, suggesting that individuals within a sampling area were more closely related than what would be predicted under a random mating model. The genetic diversity for each sampling location is included in Table S3.

**FIGURE 4:**
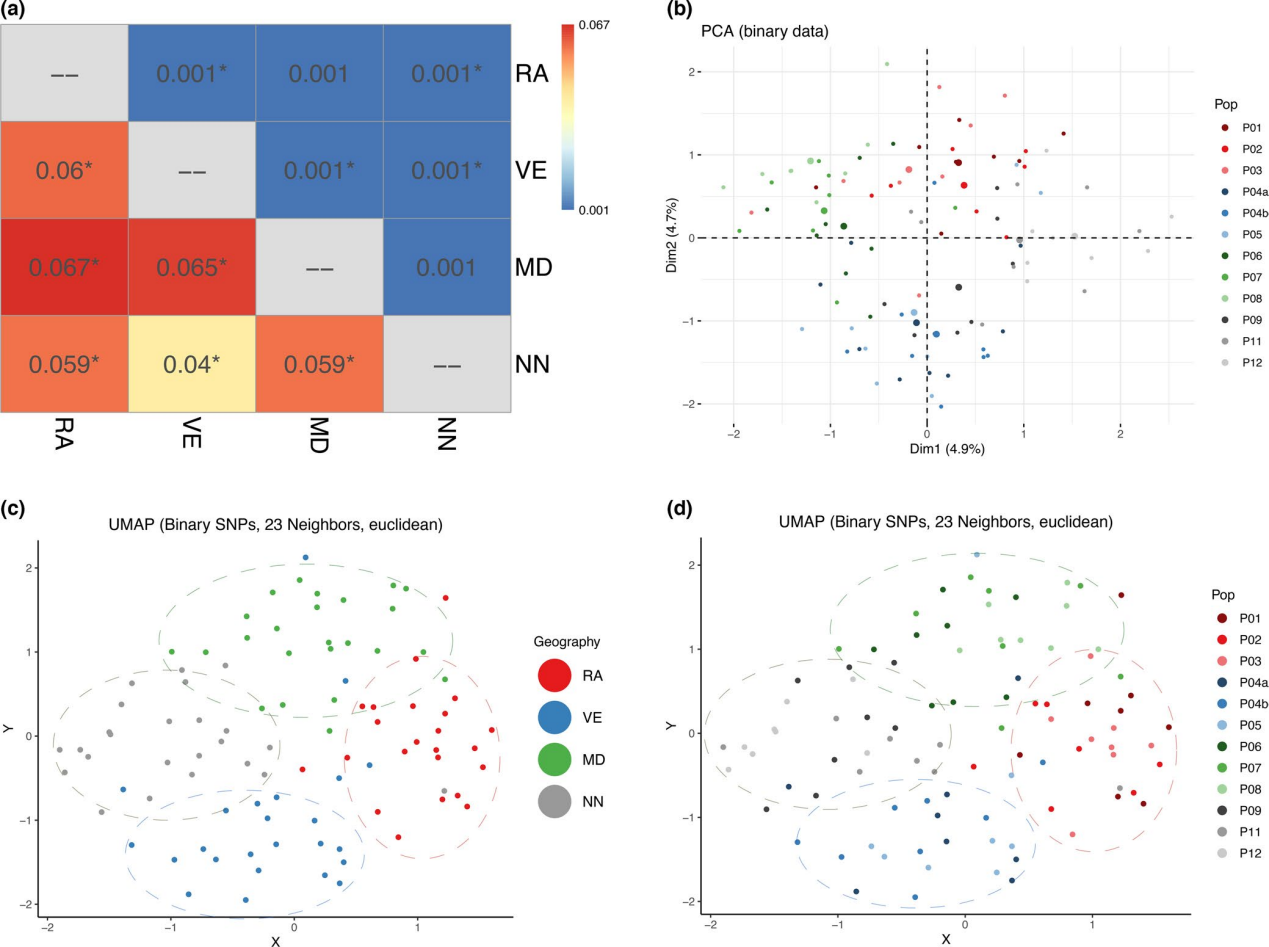
Geography‐based distinction and population structure in lice populations using Rf‐reduced datasets. (a) Heatmap showing pairwise Fst values based on Rf‐reduced (RfGeo) and full‐SNP dataset. Asterisk denotes the significant Fst values (FDR adjusted *p* < .05). The values above diagonal are the full‐SNP dataset, and the values below diagonal are the RfGeo dataset. (b) PCA plot of RfGeo dataset showing each sampling site. (c) UMAP projection of RfGeo, Euclidean distance metric, and 23 neighbors. Clusters are formed pertaining to each distinct geographical population. (d) The local populations are mixed within geographical regions clusters on C. RA: P01‐P03, VE: P04a‐P05, MD: P06‐P08, and NN: P09‐P12

Pairwise Fst was found to be weak with high gene flow, 0.001 between various geographical locations (Figure 4a) suggesting that the populations are sharing their genetic material through high levels of breeding. A significant but weak population structure with 99.6% of variation within individuals and 0.1% variance among population was observed from AMOVA (Fst = 0.001, *p* < .0001) (Table 2) suggesting a panmictic population. The Mantel test for isolation by distance indicated no significant correlation between genetic differentiation and geographical distance (Mantel *r* = .460, *p* = .045). A negative value of Tajima's D was calculated, indicating an excess of low‐frequency polymorphisms relative to expectation (Tajima's *D* = −3.425931, *p* < .001). No population structure for geographical areas was observed using PCA, and no clusters were formed for sex differentiation. The visualization using UMAP did not show any clustering for the geographical locations and sex differentiation (Figures S2 and S3).

**TABLE 2:** Analysis of molecular variance showing the global population structure

Dataset	Source of variation	Nested in	%var	*F*‐stat	*F*‐value	*p*‐value
Full SNP	Within Individual	‐‐	99.6	F_it	0.004	‐‐
	Among Individual	Population	0.3	F_is	0.003	.009
	Among Population	‐‐	0.1	F_st	0.001	.001
Rf‐reduced SNPs	Within Individual	‐‐	89	F_it	0.11	‐‐
	Among Individual	Population	4.8	F_is	0.051	.001
	Among Population	‐‐	6.2	F_st	0.062	.001

### 3.5 Using machine learning to define population structure and sexual differentiation

The supervised machine learning technique Random Forest was used to detect important SNPs for population structuring. We reduced the dataset dimension based on the regional geography (Geo) and gender‐disparity (Sex) features. For geography, a subset of 91 SNPs (RfGeo) was identified, and a model built on these SNPs was able to classify each sample's originating region with an accuracy of 43.75%. Similarly, a sex‐specific dataset of 14 SNPs (RfSex) was derived and was used to correctly classify the sex with 87.5% accuracy on the unseen data. These reduced datasets with highly discriminatory loci were used to conduct downstream analyses that were similar to those conducted on the full‐SNP dataset.

**FIGURE 5:**
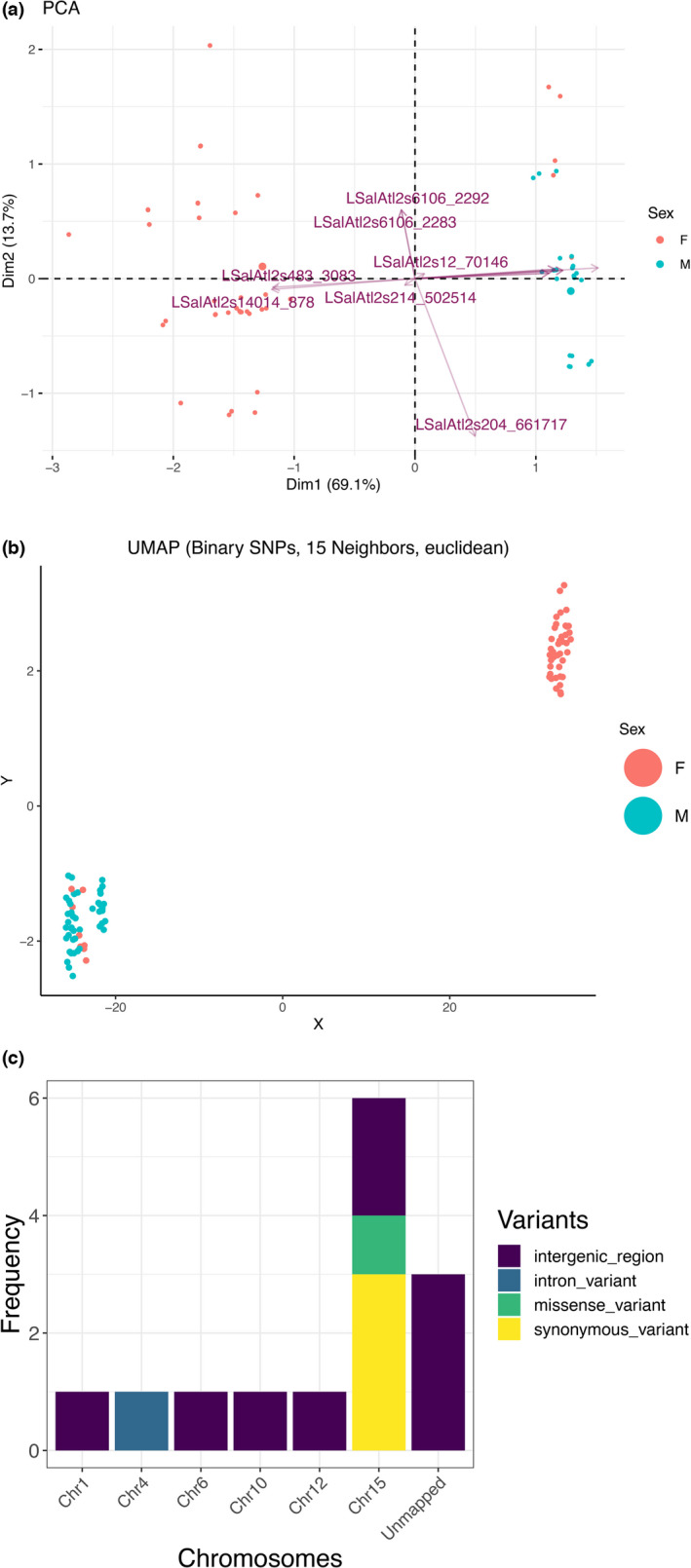
Sex‐based distinction mapping of sex‐specific SNPs at the chromosomal level. (a) PCA biplot of RfSex dataset where blue dots represent male individuals and pink dots represent females. The arrows for each SNPs point in the direction of increasing values of that variable. (b) UMAP projection of RfSex, euclidean metrics, and 13 neighbors. Two distinct clusters are formed, one for each sex. (c) RfSex SNPs were mapped across the chromosomes using the IoA‐001 assembly. In the chromosomal assembly, 11 SNPs were mapped out of 14. Variant types are represented by distinct colors, and a number of mapped variants per chromosome are shown

**FIGURE 6:**
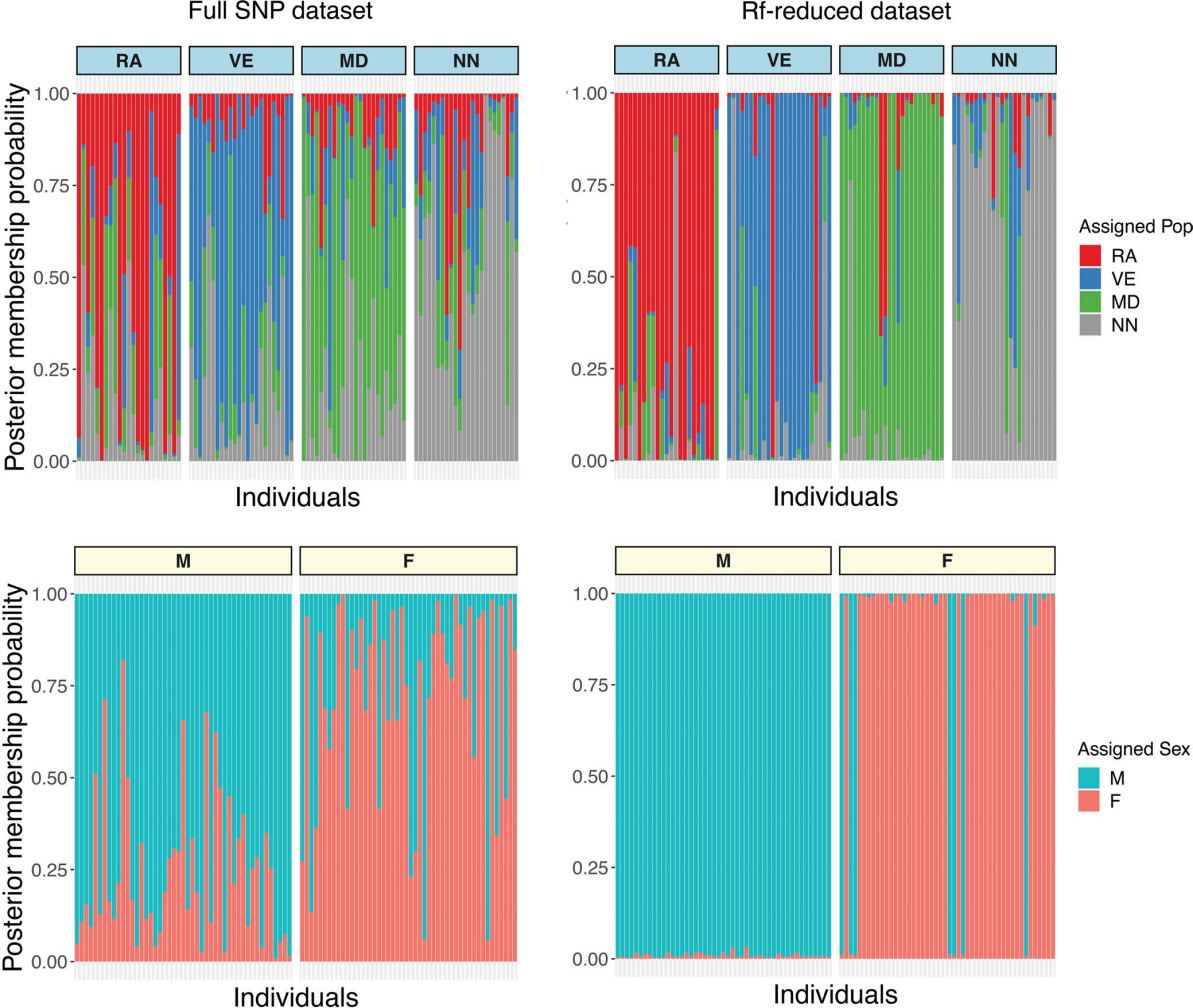
The membership probability plot showing the probability of each individual being assigned to a population or sex. Membership probability plots for population assignment in geographical regions using DAPC, where each bar represents a single individual. Upper panel: Full‐SNP dataset (left) and important SNPs RfGeo (left). Membership probability plot for sex assignment, where each bar represents a single individual. Lower panel: Full‐SNP dataset (left) and important SNPs RfSex (right)

PCA performed on the RfGeo dataset revealed a greater spatial distinction between the regional populations (Figure 4b). When compared to the entire dataset (Figure S3), the sex‐based distinction with RfSex was clearly defined (Figure 5a). The population assignment analysis with discriminant analysis principal components (DAPC) showed a mean population assignment accuracy of 0.77, and most individuals were correctly allocated to geographical regions, compared with a 0.56 accuracy for the full dataset. Similarly, the mean sex assignment accuracy increased to 0.91 for RfSex from 0.73 for the entire dataset. The membership plots of population and sex assignment are presented in Figure 6.

The pairwise comparisons of Fst values between the full‐SNP dataset with the RF‐reduced dataset based on geographical regions (geography) are shown as a heatmap (Figure 4a). Pairwise comparisons of Fst showed highly significant (FDR adjusted *p* < .01) values ranging from 0.04 to 0.067 suggesting a considerable degree of differentiation within geographical regions. Additionally, the pairwise difference for population in each sampling site (P01–P12) is shown as a heatmap (Figure S4). Similarly, the output of AMOVA with RfGeo, source of variation within individual was 89% and variance among the population was 6.2% (Fst = 0.06, *p* < .001), suggesting a population structure of salmon lice. Results from UMAP analysis showed that it was possible to distinguish between geographical regions for RfGeo (Figure 4c), and local populations were mixed within geographical clusters (Figure 4d). The euclidean distance metric was able to better capture the discrepancies for larger numbers of neighbors (*n* >= 10). Likewise, the gender‐specific clustering was strongly observable using the RfSex dataset with both PCA and UMAP methods (Figure 5a,b).

### 3.6 Annotation of the SNPs

We investigated the regions upstream and downstream of the outlier SNPs in the annotated genome to classify genes possibly involved in local adaptation and under positive selection in salmon lice. We also used the *Boruta* algorithm (Kursa & Rudnicki, 2010) to classify the SNPs that are of higher importance among the 91 SNPs identified (Figure S5). One of the eighteen SNPs was located upstream of the gene variant encoding a protein orthologous to the *Drosophila melanogaster* corepressor splits ends (Spen). Four of the SNPs were found to be intron variants. The rest of the SNPs were between intergenic regions of different genes. Table 3 summarizes the outlier SNPs and annotation.

**TABLE 3:** Annotation of outlier SNPs from various genome‐scan methods and highly ranked SNPs from random forest

Contig_position	Locus	SNP	Bayescan	Arlequin	Rf	Annotation
LSalAtl2s18_783602	625	T:C	No	No	Yes	intergenic_region
LSalAtl2s41_211832	4,443	C:G	No	No	Yes	intron_variant
LSalAtl2s60_347705	7,031	T:C	No	Yes	No	intergenic_region
LSalAtl2s60_347724	7,032	G:A	No	Yes	No	intergenic_region
LSalAtl2s70_134344	7,330	C:T	No	No	Yes	intron_variant
LSalAtl2s116_626766	434	C:T	No	No	Yes	intron_variant
LSalAtl2s140_542836	9,380	G:A	No	Yes	No	intergenic_region
LSalAtl2s166_420680	6,551	T:C	No	No	Yes	intergenic_region
LSalAtl2s194_195505	5,624	T:G	Putative	Yes	Yes	intergenic_region
LSalAtl2s267_411921	11,550	T:A	No	No	Yes	intergenic_region
LSalAtl2s301_279372	7,303	C:T	No	Yes	Yes	intergenic_region
LSalAtl2s359_216742	10,150	C:A	No	Yes	No	intergenic_region
LSalAtl2s359_216752	10,151	A:C	No	Yes	No	intergenic_region
LSalAtl2s443_387864	9,078	C:T	No	No	Yes	intron_variant
LSalAtl2s663_572884	4,414	C:G	No	Yes	Yes	upstream_gene_variant: Split ends like
LSalAtl2s881_180761	15,067	C:A	No	Yes	Yes	intergenic_region
LSalAtl2s976_75870	16,465	C:T	No	No	Yes	intergenic_region
LSalAtl2s2627_5290	17,953	C:G	Putative	Yes	No	intergenic_region

We have also outlined and annotated the important SNPs in sex determination (Table 4). The SNPs were mapped across the chromosome using IoA‐00 assembly (Figure 5c). Four SNPs were annotated as synonymous or missense. We have identified two SNPs in the coding region of subunit 8 of the general transcription factor TFIID. In addition, two SNPs were found to be in the coding region of the hypothetical protein fumble/pantothenate kinase.

**TABLE 4:** Annotation of SNPs associated with sex determination

Contig_position	SNP	Annotation
LSalAtl2s12_70146	A:T	intron_variant: EMLSAG00000001766
LSalAtl2s204_661717	G:T	intergenic_region
LSalAtl2s214_502514	G:T	intergenic_region
LSalAtl2s483_3083	G:A	intergenic_region
LSalAtl2s5137_4777	A:T	missense_variant: EMLSAG00000008398 Transcription initiation factor TFIID subunit 8
LSalAtl2s5137_4780	T:C	synonymous_variant: EMLSAG00000008398 Transcription initiation factor TFIID subunit 8
LSalAtl2s6106_2283	T:A	intergenic_region
LSalAtl2s6106_2293	C:T	intergenic_region
LSalAtl2s14014_878	G:A	intergenic_region
LSalAtl2s14597_218	A:T	intergenic_region
LSalAtl2s20805_361	G:A	synonymous_variant: EMLSAG00000003836 pantothenate kinases or fumble/ hypothetical
LSalAtl2s20805_370	T:C	synonymous_variant: EMLSAG00000003836 pantothenate kinases or fumble/ hypothetical
LSalAtl2s24901_228	C:A	Intergenic region
LSalAtl2s24901_248	A:G	Intergenic region

## 4 DISCUSSION

We used IIb‐RAD sequencing technique in conjunction with advanced population genetic analyses and machine learning methods to study *L*. *salmonis* populations in coastal geographical locations along Norway and to identify potential markers of selection. Despite the significant effect of salmon lice on Norwegian aquaculture industry, few studies have been conducted to determine the importance of population genetics in salmon lice management. There is also gap in the knowledge of the sexual dimorphism in salmon lice. Sexual dimorphism, the state in which there is a difference between individuals of different sex other than that of morphology of sexual organs, delineates the critical components of higher eukaryotic biology (Seale et al., 2018). We have investigated the population genetic structure of lice and differences between sexes, and their importance.

### 4.1 Sample size and selection of Random Forest

A limitation in genetic studies is the small number of samples available and the higher number of associated markers. The cost of producing sequencing data from a larger number of samples is enormous, which is challenging with limited budget available. A study on invasive whitefly suggested that a sample size of more than four individuals had very little impact on genetic diversity estimates (Qu et al., 2019). A 2018 study in salmon lice also employed a sample size of 11–14 individuals in each region for a similar investigation in the North Atlantic (Jacobs et al., 2018). In this study, the samples included a total of 95 salmon lice individuals. In each site, 11–12 individuals were collected, with each region comprising 23–24 individuals.

Genomic data contains numerous noninformative markers and removing them is critical. We used Random Forest to analyze the data and removed such uninformative markers using the backward purging approach described elsewhere (Brieuc et al., 2018; Holliday et al., 2012). Random forest is a powerful machine learning approach that can analyze large genomic datasets to discover loci underlying polygenic traits in circumstances when sample size is small and number of markers is large (Brieuc et al., 2018; Qi, 2012). Other studies have used and described a generalization of the backward purging approach (Holliday et al., 2012), to eliminate such noninformative markers (Brieuc et al., 2015, 2018; Waters et al., 2018). However, backward purging may increase the likelihood of overfitting, which occurs when RF fits noise inside the training data set (Díaz‐Uriarte & Alvarez de Andrés, 2006; Jiang et al., 2004). Overfitting becomes more prevalent as the number of loci included in association analyses increases. It may result in the inclusion of false positives within the final set of predictor loci and a decrease in prediction accuracy for new samples (Wray et al., 2013). To address this, cross‐validation was used. The data were split into training and test datasets, and the model's performance was evaluated using the test dataset.

### 4.2 Population structure

Norwegian waters are vital for salmonid aquaculture, and an understanding of the genetic connectivity of salmon lice among geographically proximate population is therefore of particular importance. Using a random forest‐based approach, we identified the loci for geography‐based distinction of salmon lice against a backdrop of high genetic connectivity. We have also observed the overlap between random forest identified loci and the loci under positive selection from genome‐scan approaches. Salmon lice spreads quickly across aquaculture sites as a result of dispersal, hydrodynamic fluctuation over the spatiotemporal scales, via the migratory and resident host salmonids, and salmonids transportation by commercial well vessels (Boxaspen, 2006; Johnsen et al., 2016; Morton et al., 2011; Salama et al., 2013). These factors of spreading raise the possibility of salmon lice mixing across different populations, resulting in lower genetic diversity in case of negative selection. Previous studies have suggested that comparable findings can be obtained with a high degree of dispersal (Glover et al., 2011; Nolan & Powell, 2009; Todd et al., 2004). Studies of marine organisms have revealed that population genetic structure is poor or nonexistent at large geographical scale, possibly due to large population sizes, high fertility and dispersal potential, and small size of larvae carried over longer distances by wind and sea currents (Palumbi, 2003; Truelove et al., 2017; Ward et al., 1994; White et al., 2010). A recent analysis of the structure of the salmon lice population along the North Atlantic (Ireland, Scotland, and Norway) uncovered a significant population structure using IIb‐RAD sequencing in conjunction with a random forest classification approach (Jacobs et al., 2018). In our study, we have targeted to identify the genetic population structure of salmon lice along the Norwegian coastline.

According to our findings, there is a major regional population distinction among salmon lice populations. However, there was a relative weak association of genetic variation with geographical distance (Mantel *r* = 0.4601, *p* = .045), which is similar to the previous studies, and genetic drift had little impact on population structure (Besnier et al., 2014; Jacobs et al., 2018; Nolan & Powell, 2009; Todd et al., 2004). The absence of isolation by distance may be attributed to a variety of reasons, including high dispersal of larvae and wild‐salmon migration (Besnier et al., 2014). Given that genetic drift is not a major driving factor, gene flow may be the driving force behind heterogeneity observed in previous studies (Johnsen et al., 2016; Salama et al., 2013). Natural selection may have an impact on population structure due to the short life span of the parasite, and the use of the same anti‐parasitic drugs for salmon lice management, which may affect multiple genomic regions of salmon lice, could present selective pressure (Besnier et al., 2014; Coates et al., 2021; Costello, 2006; Fjørtoft et al., 2020; Messmer et al., 2018). According to the Norwegian Veterinary Institute, the susceptibility of salmon lice to anti‐parasitic medications varies along the Norwegian coast, as does the prevalence of medical treatments in different regions (Helgesen et al., 2020, 2021). Tajima's D was negative, suggesting an abundance of rare alleles, implying a recent selective sweep or linkage to swept genes. Linkage groups 1 and 5 especially demonstrated signs of selective sweeps, with linkage group 5 associated with drug resistance in previous studies (Besnier et al., 2014; Kaur et al., 2017). The primary cause of differential selection across populations is most likely spatial‐temporal variation in drug resistance, but local environmental variables may also act as additional selective pressures driving allele frequency disparities between populations (Jacobs et al., 2018). Furthermore, non‐medical treatments such as freshwater, thermal, and local environmental factors that affects salmon lice survival and development, such as seawater temperature and salinity, and host density could also contribute to selective pressure (Bricknell et al., 2006; Coates et al., 2021; Ljungfeldt et al., 2017; Mennerat et al., 2017; Powell et al., 2015; Wright et al., 2016). Anti‐parasitic drugs and non‐medical treatment methods present external pressure on the salmon lice population. Since parasites are highly adaptable organisms, they adapt to such pressures driving the evolution and generation of variants (Coates et al., 2021). In accordance with previous studies, our study revealed that use of a machine learning approach based on random forest classification increased the resolution of population structure in a species with high gene flow and low genetic diversity (Jacobs et al., 2018; Laporte et al., 2016).

Quantitative traits of marine organisms have a polygenic genomic basis, and our findings and previous research suggest that the adaptive phenotype of salmon lice against medical treatments and the local environment has a polygenic genomic basis (Berg & Coop, 2014; Jacobs et al., 2018). Genetic diversity is principally divided into adaptive and neutral. A large portion of the genome is neutral, referring to the portion of the gene or locus that has little impact on fitness but rather informs us about the gene flow, migration or dispersal (Holderegger et al., 2006). In our study, a significant portion of the loci involved in population structuring were located in the neutral region, highlighting the significance of neutral genomic regions. Most of the loci under putative selections were found between the neutral intergenic regions. We have found a possible selection marker that is upstream gene variant SNP of protein split ends (Spen). The next step is to identify and classify the genes that are under positive selection in order to reveal the selective pressure for population structure.

### 4.3 Sex‐specific differentiation

The dataset of SNPs was also used to differentiate the male and female populations of salmon louse. RfSex, a subset of the original SNP dataset was generated using the random forest classification and comprised of 14 SNPs. These SNPs were used to distinguish male from female individuals in the population using dimension reduction approaches such as PCA and UMAP, allowing for an optimal visualization of the respective data clusters. The formation of separate clusters for each sex, validated the use of random forest in the search for significant SNPs in sex categorization. However, eight females were incorrectly assigned genetically as males. Since pre‐adult life stages were used for the study, pre‐adult males could have been selected erroneously as females during morphological identification resulting in the misassignment.

The SNPs were also annotated according to the highest importance ranking and variant impact. We found the presence of one missense and one synonymous mutation in the protein encoding gene TFIID subunit 8. Two synonymous mutations in Pantothenate kinase/fumble were also identified with high importance for sex classification. These indicators could be possible sex markers, and these genes could play a distinct role in males and females. Pantothenate kinase is involved in the conversion of pantothenate to CoA, which is required for the synthesis of several types of essential lipids and in the TCA cycle (Sakae & Tanaka, 2021). Inhibition of the pantothenate metabolic pathway in starved medaka (*Oryzias latipes*) was recently linked to sex reversal from female to male (Sakae et al., 2020). Furthermore, pantothenate kinase is expressed twice as much in adult females than in adult males (LiceBase, 2021). Although synonymous mutations were previously assumed to be silent, they may influence protein activities by disrupting the post‐transcriptional processing and regulation of RNA (Sauna & Kimchi‐Sarfaty, 2011). Similarly, the role of the transcription factor TFIID in mammals has been characterized, and a gonad‐specific component of TFIID has been found to be enriched in the mouse testis, which is necessary for the maintenance of spermatogenesis (Falender et al., 2005). It may be interesting to investigate the significance of these two genes in salmon lice and see whether there are any variations in how they function in different sexes.

There have been few attempts to study the sex related genes in salmon lice. Earlier, a sex‐linked SNP marker in the coding region of the prohibitin‐2 gene was discovered in *L*. *salmonis* (Carmichael et al., 2013) and *Caligus rogercresseyi* (Farlora et al., 2015), which was homozygous in males and heterozygous in females. This was also consistent with recent findings that female heterogamety is the sex determinant in salmon lice with both ZZ/ZW chromosome system (Danzmann et al., 2019; Skern‐Mauritzen et al., 2021). In agreement with earlier studies, chromosome 15 or linkage group 15 contains the greatest number of sex‐specific SNPs, suggesting that it is a sex chromosome (Danzmann et al., 2019; Skern‐Mauritzen et al., 2021). Furthermore, chromosome 15 is considerably smaller than other chromosomes, about one‐third the size, yet it has a significant number of sex‐specific SNPs identified by random forest. The investigation of genes identified as having essential SNPs for sexual differentiation may be valuable for understanding the molecular mechanisms underlying sex‐specific processes in *L*. *salmonis*. Apart from genes, there are significant SNPs in the neutral regions of the genome that were chosen as essential markers for sexual differentiation, also demonstrating the importance of neutral genomic regions in sex bias.

## 5 CONCLUSION

The focus of our study was to use IIb‐RAD sequencing jointly with a random forest classification algorithm to detect the population structure in the Norwegian salmon lice population. In addition, we identified SNP markers that could be important for differentiating between male and female salmon louse. We were able to classify regional demographic clusters of salmon lice populations along the Norwegian coast. Further research is needed to determine whether diverse populations respond differently to various delousing approaches. Our study may contribute to the development of novel tailored methods, intervention strategies and management of an economically significant pest.

## CONFLICT OF INTEREST

The authors declare no conflicts of interest.

## AUTHOR CONTRIBUTIONS

**Prashanna Guragain:** Conceptualization (lead); Data curation (lead); Formal analysis (lead); Investigation (lead); Methodology (lead); Resources (equal); Validation (lead); Visualization (lead); Writing – original draft (lead); Writing – review & editing (equal). **Anna Solvang Båtnes:** Conceptualization (supporting); Funding acquisition (equal); Project administration (lead); Supervision (equal); Validation (supporting); Writing – review & editing (equal). **John Zobolas:** Data curation (supporting); Software (equal); Visualization (equal); Writing – review & editing (equal). **Yngvar Olsen:** Funding acquisition (lead); Project administration (supporting); Resources (equal); Supervision (equal); Validation (equal); Writing – review & editing (equal). **Atle M. Bones:** Conceptualization (equal); Funding acquisition (equal); Resources (lead); Supervision (equal); Validation (equal); Writing – review & editing (equal). **Per Winge:** Conceptualization (lead); Data curation (equal); Formal analysis (equal); Funding acquisition (equal); Investigation (equal); Methodology (equal); Project administration (equal); Resources (equal); Software (equal); Supervision (lead); Validation (equal); Visualization (supporting); Writing – review & editing (equal).

### OPEN RESEARCH BADGES

This article has been awarded Open Materials, Open Data, Preregistered Research Designs Badges. All materials and data are publicly accessible via the Open Science Framework at https://www.ncbi.nlm.nih.gov/bioproject/PRJNA762270; https://doi.org/10.5061/dryad.p8cz8w9r9; https://github.com/g3prashan/IIb‐RADseq_popgen.

## Supporting information

Supplementary Material

## Data Availability

The raw data is available at NCBI SRA database with BioProject PRJNA762270. https://www.ncbi.nlm.nih.gov/bioproject/PRJNA762270. The data used for this article are available from Dryad. https://doi.org/10.5061/dryad.p8cz8w9r9 (Guragain, Båtnes, et al., 2021) .These files include. File 1. Full‐SNP data containing 19428 SNPs. File 2. RfGeo, essential 91 SNPs. File 3. RfSex, essential 14 SNPs. The code used to make figures in the text is deposited into the GitHub repository and is publicly available. https://github.com/g3prashan/IIb‐RADseq_popgen
