# Supplementary material for: IIb‐RAD‐sequencing coupled with random forest classification indicates regional population structuring and sex‐specific differentiation in salmon lice (Lepeophtheirus salmonis)
Source: Ecol Evol. 2022 Apr 6;12(4):e8809. doi: 10.1002/ece3.8809 (PMC8986551; doi:10.1002/ece3.8809)
Supplement: Supplementary file 1 — Supplementary Material [file ECE3-12-e8809-s001.docx]

**Supplementary materials**

**Supplementary Table 1: Overview of sample collection sites**

|  | **Production area** | **Locality number** | **Latitude** | **Longitude** |
| --- | --- | --- | --- | --- |
| P01 | Pinnen, Flekkefjord | 11854 | 58.13398 | 6.43913 |
| P02 | Låva, Stavanger | 19355 | 59.35973 | 5.44352 |
| P03 | Hillersvik, Sveio | 10300 | 59.36977 | 5.20885 |
| P04a | Oslandsurda, Høyanger | 12179 | 61.05407 | 5.49925 |
| P04b | Ånnaholmane, Askvoll | 11793 | 61.17343 | 4.44365 |
| P05 | Voldnes, Herøy | 13246 | 62.12767 | 5.47027 |
| P06 | Aursøysva, Frøya | 12357 | 63.4833 | 8.54575 |
| P07 | Geitholmen, Nærøysund | 26335 | 64.4819 | 11.01562 |
| P08 | Øksningen, Bindal | 14019 | 65.08582 | 12.19162 |
| P09 | Gaukværøy, Bø in Nordland | 38757 | 68.36985 | 14.2082 |
| P11 | Nøklan, Kvænangen | 15659 | 69.54687 | 21.50097 |
| P12 | Tinnlandet, Hammerfest | 15517 | 70.31987 | 23.13757 |

**Supplementary Table 2: Annotations of mutations with high functional level impact**

| **Contig_position** | **SNP** | **Variant** | **Gene** |
| --- | --- | --- | --- |
| LSalAtl2s139_1358506 | C:A | stop_gained | EMLSAG00000002137 |
| LSalAtl2s142_629730 | G:T | stop_gained | EMLSAG00000002273 |
| LSalAtl2s240_67106 | G:C | splice_acceptor_variant  &intron_variant | EMLSAG00000004625 |
| LSalAtl2s62_295650 | G:C | stop_gained | EMLSAG00000009754 |
| LSalAtl2s730_283650 | G:T | stop_gained | EMLSAG00000010925 |
| LSalAtl2s455_43470 | C:G | stop_gained | EMLSAG00000007779 |
| LSalAtl2s1051_9043 | T:G | splice_donor_variant  &intron_variant | EMLSAG00000000353 |
| LSalAtl2s1791_10479 | C:T | splice_donor_variant  &intron_variant | EMLSAG00000003258 |
| LSalAtl2s4002_2662 | C:T | stop_gained | EMLSAG00000007105 |

**Supplementary Table 3: Summary statistics of each sampling locations, namely observed heterozygosity (Ho), expected Heterozygosity (Hs) and coefficient of inbreeding (Gis).**

| **Sampling location** | **Full SNPs** | | | **Rf-reduced SNPs (RfGeo)** | | |
| --- | --- | --- | --- | --- | --- | --- |
|  | **Ho** | **Hs** | **Gis** | **Ho** | **Hs** | **Gis** |
| P01 | 0.198 | 0.191 | -0.038 | 0.281 | 0.287 | 0.019 |
| P02 | 0.152 | 0.175 | 0.132 | 0.178 | 0.245 | 0.273 |
| P03 | 0.187 | 0.188 | 0.006 | 0.246 | 0.246 | -0.000 |
| P04a | 0.192 | 0.189 | -0.019 | 0.244 | 0.254 | 0.041 |
| P04b | 0.186 | 0.186 | -0.001 | 0.263 | 0.277 | 0.049 |
| P05 | 0.185 | 0.187 | 0.011 | 0.253 | 0.280 | 0.097 |
| P06 | 0.183 | 0.183 | -0.001 | 0.238 | 0.259 | 0.083 |
| P07 | 0.209 | 0.196 | -0.065 | 0.276 | 0.273 | -0.010 |
| P08 | 0.193 | 0.19 | -0.015 | 0.235 | 0.254 | 0.072 |
| P09 | 0.164 | 0.176 | 0.066 | 0.208 | 0.242 | 0.139 |
| P11 | 0.177 | 0.185 | 0.041 | 0.246 | 0.277 | 0.110 |
| P12 | 0.195 | 0.188 | -0.039 | 0.292 | 0.275 | -0.059 |


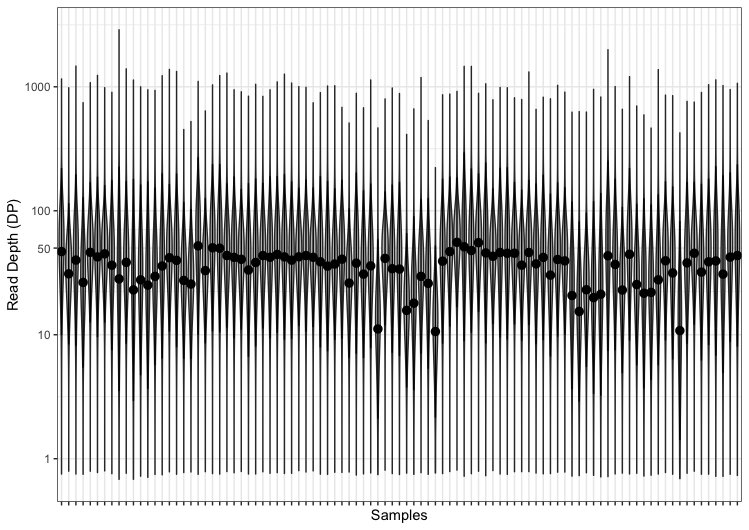


**Supplementary Figure 1: Read depth of SNPs.** The filtered depth of coverage for each individual are plotted as violin plot.

**A**

**B**

**Supplementary Figure 2: PCA and UMAP of full SNP dataset.** A. PCA plot of full SNP dataset showing each geographical area. **B**. UMAP projection, Euclidean distance metric and 23 neighbors. There are no specific clusters pertaining to geographical population.

**A**

**B**

**Supplementary Figure 3: PCA plot and UMAP of full SNP dataset for visualization of sex.** A. PCA plot of full SNP dataset showing each sex. **B**. UMAP projection, Euclidean distance metric and 15 neighbors. There are no specific clusters pertaining to sex.

**Supplementary Figure 4: Heatmap of pairwise difference based on Fst values for each sampling site.** Heatmap showing pairwise Fst values based on Rf-reduced (RfGeo) and full SNP dataset. Asterisk denotes the significant Fst values (FDR adjusted p < 0.05). The values above diagonal are full SNP dataset and the values below diagonal are RfGeo dataset.

**Supplementary Figure 5: The box plot of SNP attributes and their importance.** Boruta algorithm was used in RfGeo dataset to predict the importance **of** SNPs. SNPs having green boxplot shows the predictors are important. The red boxplots indicate they are rejected.
